# Supplementary material for: Calmodulin dissociates the STIM1-Orai1 complex and STIM1 oligomers
Source: Nat Commun. 2017 Oct 19;8:1042. doi: 10.1038/s41467-017-01135-w (PMC5648805; doi:10.1038/s41467-017-01135-w)
Supplement: Supplementary file 1 — Supplementary information [file 41467_2017_1135_MOESM1_ESM.pdf]

Supplementary Table 1: List of primers sequences used in this study

Primers used to prepare mCherry-Orai1	Orai1-sense: GTCTACAGATCTATGCATCCGG AGCCCGC<br>Orai1-antisense: CAGTCAGAATTCCTAGGCA TAGTGGCTGCCGG
Primers used to prepare STIM1-CFP, STIM1-GFP and STIM1-YFP	STIM1-sense: CAGTCAAGATCTATGGATGTAT GCGTCCGTCT<br>STIM1-antisense: CAGTCAGAATTCACTTCTTA AGAGGCTTCTTAAAGATTTTGA
Primers used to prepare SOAR-CFP, SOAR-GFP and SOAR-YFP	SOAR-sense: GTCTCAAGATCTATGGAGGCC CTTCAGAAGTGGCT<br>SOAR-antisense: CAGTCAGAATTCAGTTGTTG ACAATCTGGAAGCCACA
Primers used to prepare STIM1-LF-SS	STIM1-LF-sense: AAGAAGAGAAACACATCC TCTGGCACCTTCCAC<br>STIM1-LF-antisense: GTGGAAGGTGCCAGA GGATGTGTTTCTCTTCTT
Primers used to prepare CaM-CFP	CaM-sense: AGCTACCTCGAGATGGCTGAC CAACTGACTGA<br>CaM-antisense: GTCAGGATCCGACTTCGCT GTCATCATTTGT

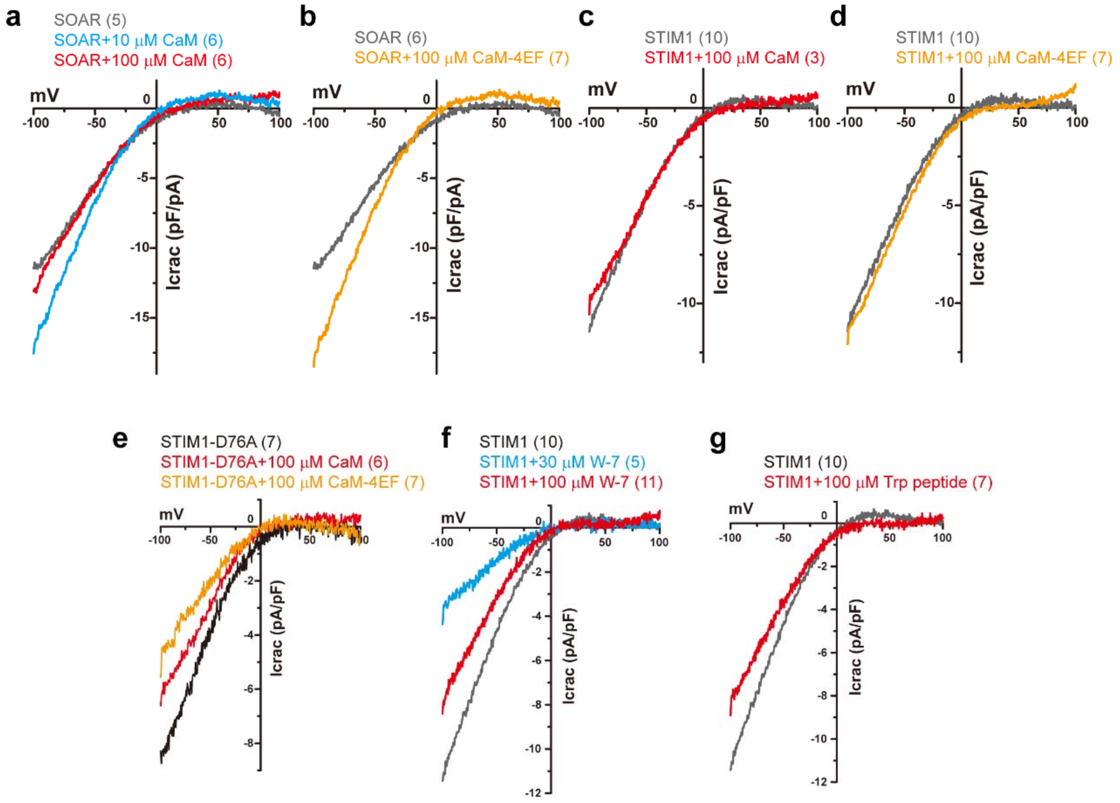

Supplementary Figure 1. Averaged current-voltage (I-V) relationship at peak current. (a) SOAR alone, SOAR with 10 μM or 100 μM CaM. (b) SOAR with or without 100 μM CaM-4EF mutant. (c) STIM1 wild type with or without 100 μM CaM. (d) STIM1 wild type with or without 100 μM CaM-4EF mutant. (e) STIM1-D76A mutant alone and with CaM wild type or CaM-4EF mutant. (f) STIM1 wild type alone and with 30 μM W-7 inhibitor or 100 μM W-7 inhibitor. (g) STIM1 wild type with or without 100 μM TRP peptide inhibitor. The I-V curves were generated from the peak current induced by the ramp stimulation protocol, representing the mean original current density sizes of individual cells. The number in the parenthesis indicates the number of cells that were analyzed.

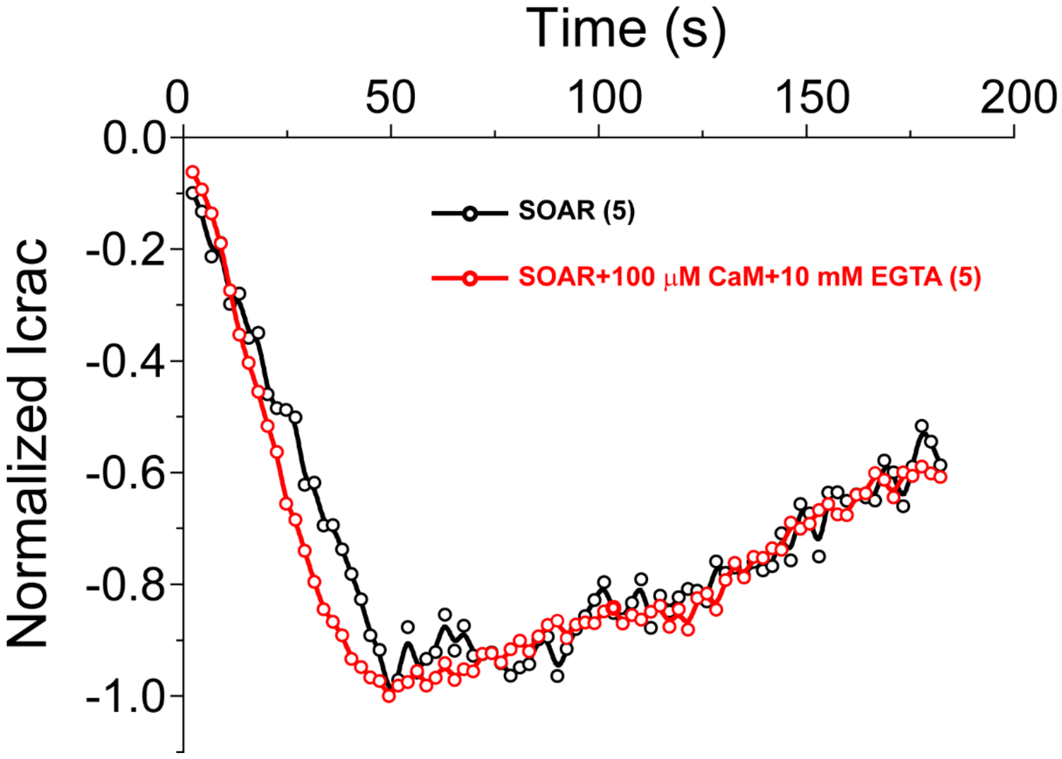

Supplementary Figure 2. High concentration of calcium chelator suppresses CaM-regulated SCDI. Normalized averaged whole cell current at -100 mV measured in HEK293 cells that express Orai1 and SOAR. In total, 10 mM EGTA and 100 μM CaM were introduced to the pipette solution. The number in the parenthesis indicates the number of cells that were analyzed.

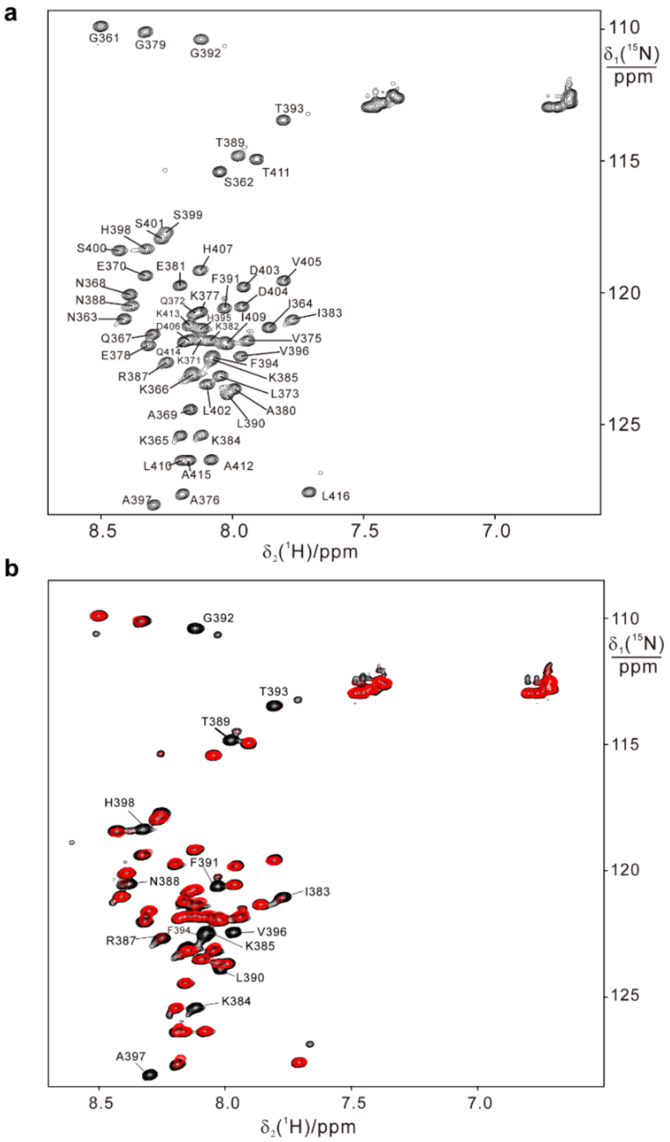

Supplementary Figure 3. Interaction of the STIM1 (363-416) construct with Ca^{2+}-CaM. (a) Peak assignment of the ^{15}N-HSQC spectrum of the STIM1 (363-416) construct. ^{15}N-HSQC spectrum of 0.2 mM ^{15}N-STIM1 in 20 mM MES (pH 6.5) and 5 mM $CaCl_2$. The spectrum was recorded at 298K at a proton frequency of 600 MHz. The residues that correspond to each backbone amide cross-peak are identified. (b) Overlay of ^{15}N-HSQC spectrum of 0.2 mM STIM1 (363-416) with (red color) or without (black color) 0.2 mM Ca^{2+}-CaM. The STIM1 residues that displayed significant intensity attenuation upon CaM binding are labeled.

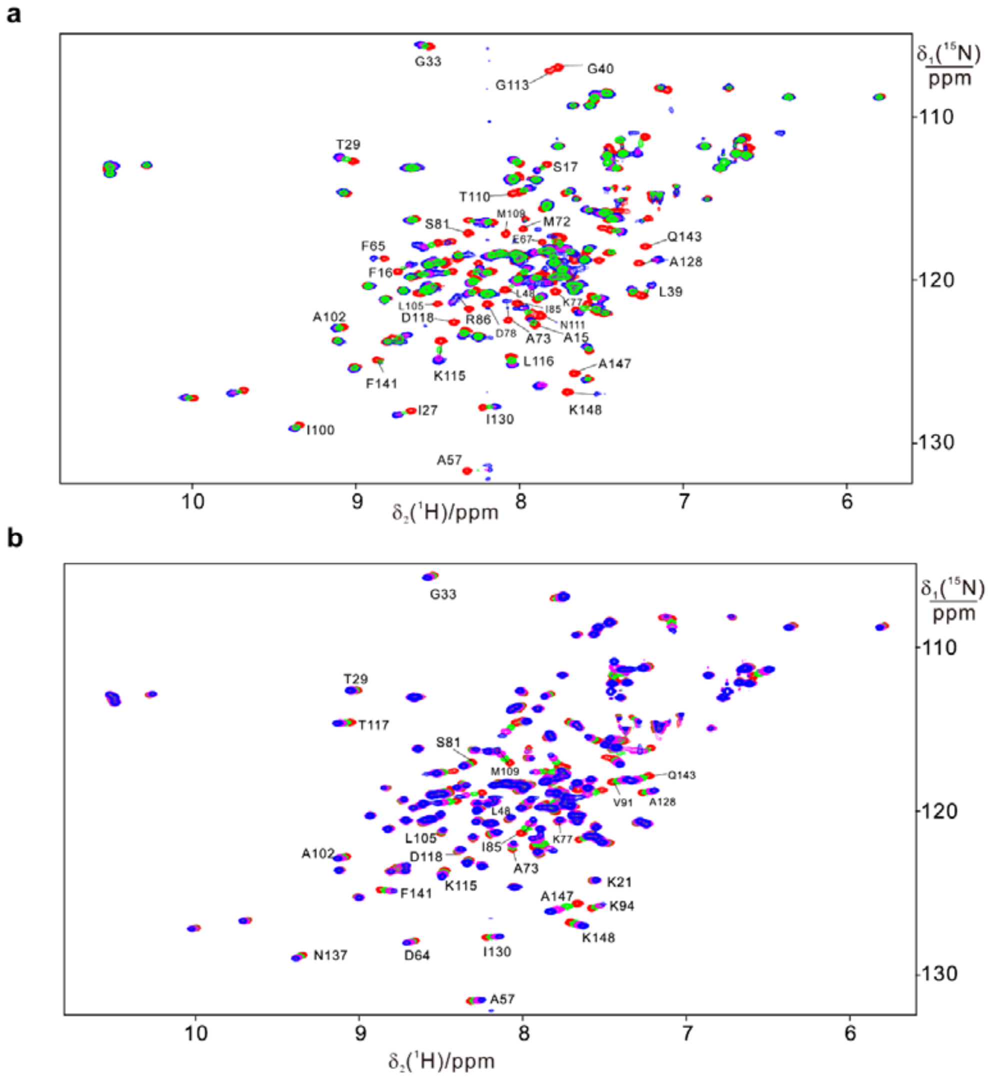

Supplementary Figure 4. Interaction of the ^{15}N-CaM-Ca^{2+} and STIM1 (363-416). (a) Overlay of ^{15}N-HSQC spectra of 0.10 mM ^{15}N-CaM-Ca^{2+} with various concentrations of STIM1 (363-416): 0 mM (red), 0.04 mM (green), 0.08 mM (magenta) and 0.12 mM (blue). The CaM residues that experienced significant chemical shift changes or intensity attenuation are labeled. (b) Overlay of ^{15}N-HSQC spectra of 0.10 mM ^{15}N-CaM-Ca^{2+} with various concentrations of STIM1 (363-416) L390S-F391S: 0 mM (red), 0.04 mM (green), 0.10 mM (magenta) and 0.20 mM (blue). The CaM residues that experienced significant chemical shift changes are labeled.

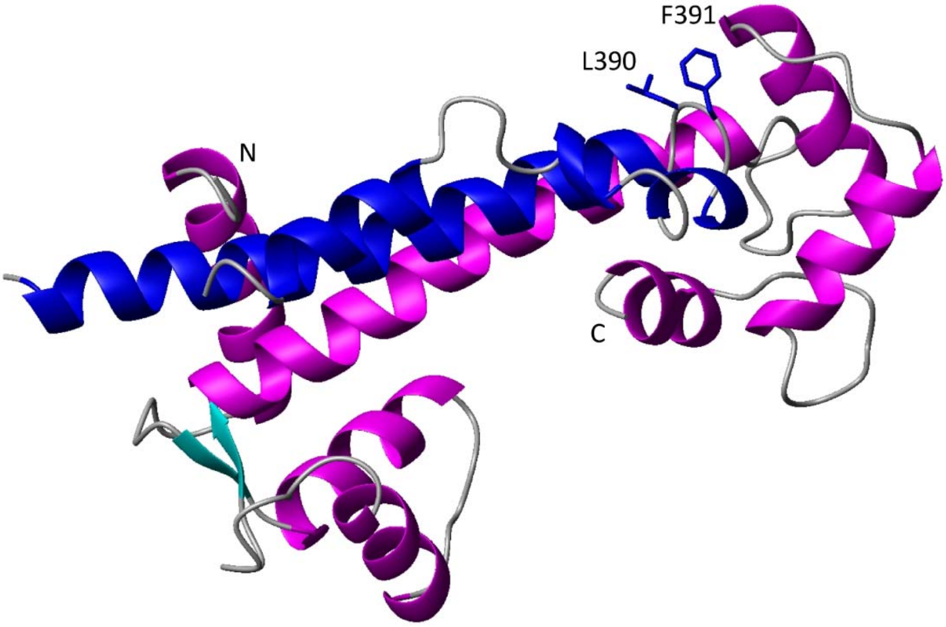

Supplementary Figure 5. Model of STIM1 (363-416) with Ca^{2+}-CaM. STIM1 is colored in blue, and CaM in magenta. The sidechains of L390 and F391 are shown as sticks. The model was built using the HADDOCK program, the chemical shift perturbations of STIM1 and Ca^{2+}-CaM and the structures of Ca^{2+}-CaM (PDB code: 1EXR) and STIM1 (PDB code:3TEQ).

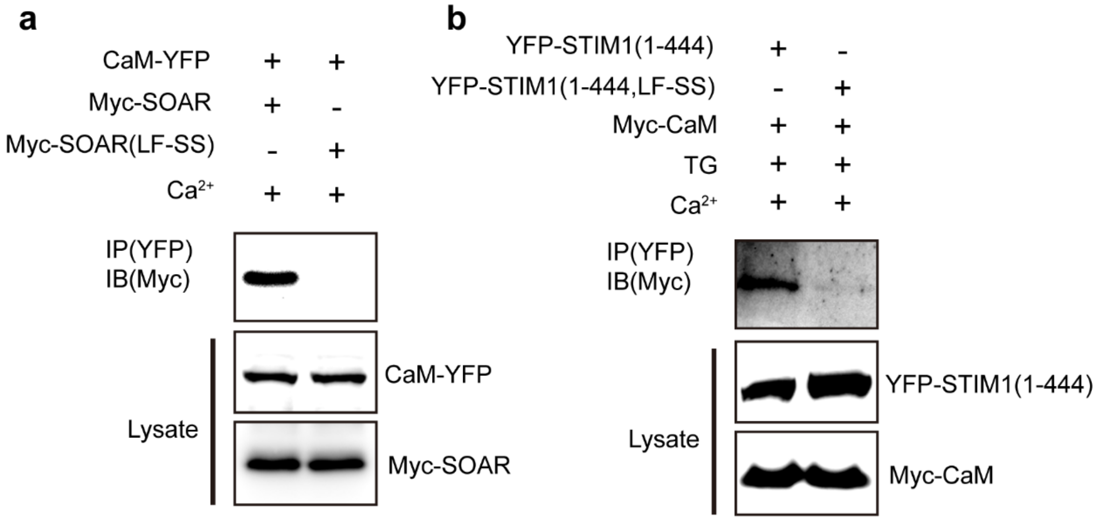

Supplementary Figure 6. Western blot analysis of co-immunoprecipitation of CaM with STIM1. (a) CaM-YFP with Myc-SOAR wild type and LF-SS mutant. (b) YFP-STIM1 (1-444) wild type and LF-SS mutant with Myc-CaM after TG treatment.

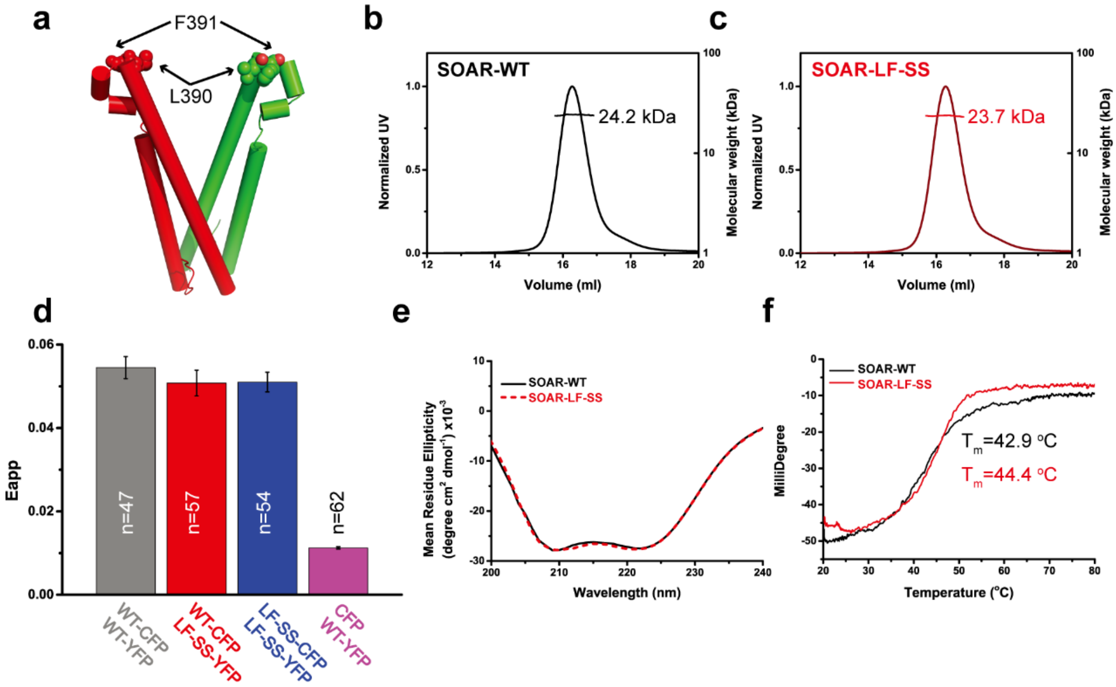

Supplementary Figure 7. Characterization of residues L390 and F391. (a) Location of residues L390 and F391 in the SOAR V-shape structure. (b, c) Multi-angle laser scattering analysis of SOAR wild type and mutant (LF-SS) proteins. (d) Resting FRET Eapp values were measured between SOAR wild type and mutant (LF-SS) in HEK293T cells. Numbers of cells that were analyzed are indicated. Error bars denote s.e.m. (e) α-Helicity of SOAR wild type and mutant (LF-SS) proteins were determined from far-UV CD spectra. (f) Thermal stability of SOAR wild type and mutant (LF-SS) proteins were determined on the basis of the ellipticity change at 222 nm from 20-80 ℃.

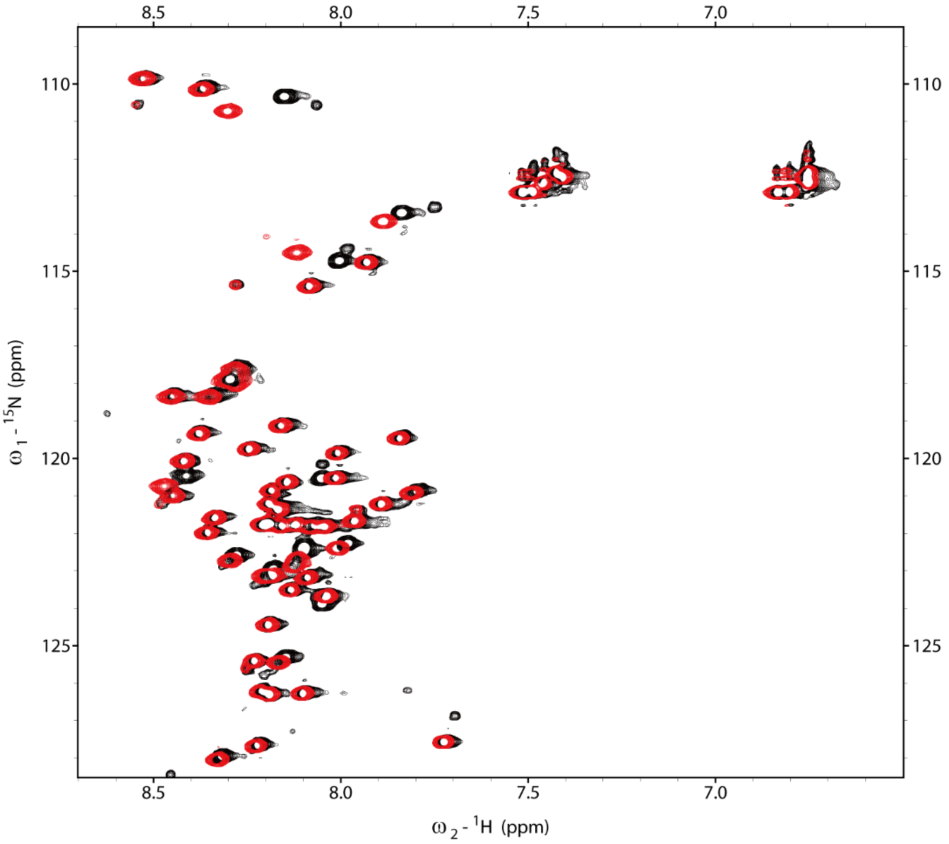

Supplementary Figure 8. SOAR wild type and L390S-F391S mutant have similar secondary structure. Overlay of ^{15}N-HSQC spectrum of 0.2 mM STIM1（363-416）wild type (black color)with ^{15}N-HSQC spectrum 0.2 mM STIM1 (363-416) L390S-F391S mutant (red color) in 20 mM MES (pH 6.5) and 5 mM $CaCl_2$.

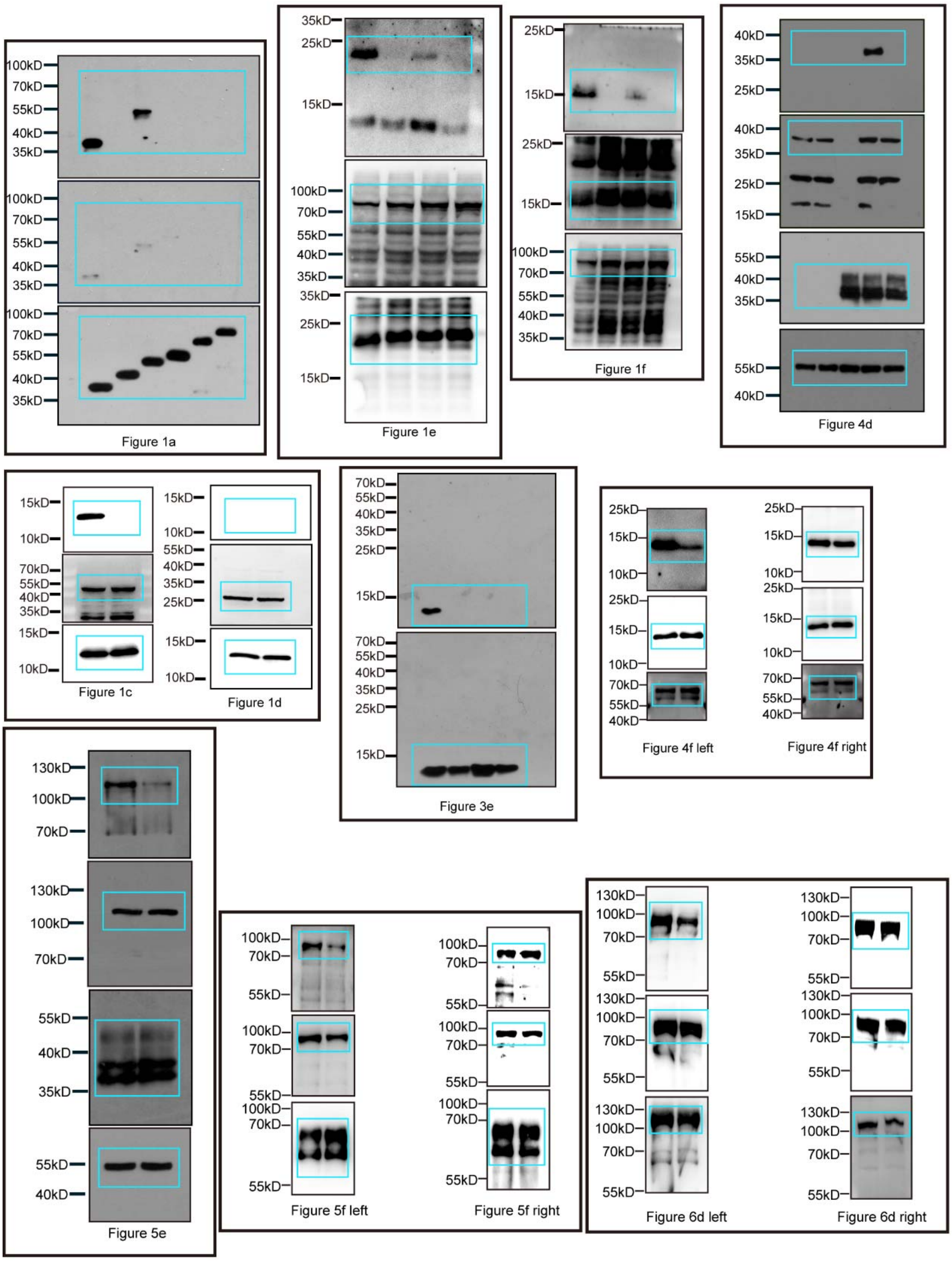

Supplemental Figure 9. Uncropped western blots.
